# Highly efficient image navigator based 3D whole‐heart cardiac MRA at 0.55T

**DOI:** 10.1002/mrm.30316

**Published:** 2024-10-16

**Authors:** Carlos Castillo‐Passi, Karl P. Kunze, Michael G. Crabb, Camila Munoz, Anastasia Fotaki, Radhouene Neji, Pablo Irarrazaval, Claudia Prieto, René M. Botnar

**Affiliations:** ^1^ School of Biomedical Engineering and Imaging Sciences King's College London London UK; ^2^ Institute for Biological and Medical Engineering Pontificia Universidad Católica de Chile Santiago Chile; ^3^ Millennium Institute for Intelligent Healthcare Engineering Pontificia Universidad Católica de Chile Santiago Chile; ^4^ MR Research Collaborations Siemens Healthcare Limited Camberley UK; ^5^ School of Engineering, Department of Electrical Engineering Pontificia Universidad Católica de Chile Santiago Chile; ^6^ Institute for Advanced Study at Technical University of Munich Munich Germany

**Keywords:** Bloch equations, Low‐field MRI, MRA, Whole‐heart

## Abstract

**Purpose:**

To develop and evaluate a highly efficient free‐breathing and contrast‐agent‐free three‐dimensional (3D) whole‐heart Cardiac Magnetic Resonance Angiography (CMRA) sequence at 0.55T.

**Methods:**

Free‐breathing whole‐heart CMRA has been previously proposed at 1.5 and 3T. Direct application of this sequence to 0.55T is not possible due to changes in the magnetic properties of the tissues. To enable free‐breathing CMRA at 0.55T, pulse sequence design and acquisition parameters of a previously proposed whole‐heart CMRA framework are optimized via Bloch simulations. Image navigators (iNAVs) are used to enable nonrigid respiratory motion‐correction and 100% respiratory scan efficiency. Patch‐based low‐rank denoising is employed to accelerate the scan and account for the reduced signal‐to‐noise ratio at 0.55T. The proposed approach was evaluated on 11 healthy subjects. Image quality was assessed by a clinical expert (1: poor to 5: excellent) for all intrapericardiac structures. Quantitative evaluation was performed by assessing the vessel sharpness of the proximal right coronary artery (RCA).

**Results:**

Optimization resulted in an imaging flip angle of $110^{\circ }$, fat saturation flip angle of $180^{\circ }$, and six k‐space lines for iNAV encoding. The relevant cardiac structures and main coronary arteries were visible in all subjects, with excellent image quality (mean 4.9/5.0) and minimal artifacts (mean 4.9/5.0), with RCA vessel sharpness (50.3%±9.8%) comparable to previous studies at 1.5T.

**Conclusion:**

The proposed approach enables 3D whole‐heart CMRA at 0.55T in a 6‐min scan (5.9±0.7 min), providing excellent image quality, minimal artifacts, and comparable vessel sharpness to previous 1.5T studies. Future work will include the evaluation of the proposed approach in patients with cardiovascular disease.

## 1 INTRODUCTION

Cardiovascular disease (CVD) is the leading cause of death globally,^1^ with nearly 80% of CVD deaths occurring in low‐ and middle‐income countries.^2^ This is due to limited accessibility to diagnostic imaging, screening tools, and sufficiently trained human resources in image acquisition and interpretation.^3^

Cardiac Magnetic Resonance Angiography (CMRA) has proven to be a key noninvasive diagnostic imaging technique to assess CVDs.^4^, ^5^ Free‐breathing three‐dimensional (3D) whole‐heart CMRA offer detailed insights into cardiac structures and the great vessels, and has demonstrated to be valuable in the assessment and follow‐up of Congenital Heart Disease,^6^, ^7^, ^8^, ^9^ Coronary Artery Disease,^10^, ^11^, ^12^, ^13^ and interventional imaging,^14^, ^15^ among others.^16^

Contrast‐enhanced (CE) CMRA techniques use contrast agents^17^ to improve the signal‐to‐noise ratio (SNR) by shortening the $T_{1}$ relaxation of the blood, which achieves a higher steady‐state signal with fewer radiofrequency (RF) excitations.^4^, ^18^ Nevertheless, there is a growing interest in non‐CE approaches^19^, ^20^ due to some concerns with the use of contrast agents, especially in populations that require repeated MRI scanning (such as patients with congenital heart disease).

Traditionally, non‐CE 3D whole‐heart CMRA is electrocardiogram (ECG)‐gated and relies on one‐dimensional diaphragmatic navigators,^21^, ^22^ accepting data acquired within a narrow respiratory gating window (usually at end‐expiration). This approach has been investigated at 1.5, 3, and 7T, and more recently at 0.55T.^23^ However, the need for data rejection results in low respiratory scan efficiency, thus leading to long and unpredictable scan times.

Multiple techniques have been proposed over the last years to overcome these challenges.^11^ Self‐navigation,^16^, ^24^ image navigators (iNAVs),^25^ and nonrigid motion corrected reconstruction techniques,^26^ combined with different undersampled reconstruction techniques have been proposed to accelerate the scan and enable 100% respiratory scan efficiency. All of these approaches have been proposed at 1.5^9^, ^27^ and 3T.^28^

The recent development of high‐end 0.55T scanners could increase the accessibility and affordability of cardiac MRI.^29^, ^30^ Furthermore, promising results have been shown in terms of patient comfort and safety, reduced, implant‐induced image artifacts and facilitation of MRI‐guided catheterizations.^31^, ^32^

The reduction in magnetic field strength leads to changes in the magnetic properties of the tissues. The most well‐known effect is the reduction in the equilibrium magnetization ($M_{0}$), and consequently SNR,^33^ which historically justified the development of higher field strength MR scanners. Despite these disadvantages, there are many advantageous physical changes that can be leveraged to recover image quality, like faster $T_{1}$ recovery, longer $T_{2}$ and $T_{2}^{*}$ decay, and decreased $B_{0}$/$B_{1}$ inhomogeneity artifacts.^30^ Offering new promising applications in regions close to high susceptibility sources.^31^, ^32^ However, non‐CE 3D whole‐heart CMRA with 100% respiratory scan efficiency has not been demonstrated at 0.55T.

The main challenges that need to be addressed to enable whole‐heart CMRA at 0.55T are lower SNR and the performance of fat suppression, since the unwanted signal from myocardial fat will recover faster at 0.55T than at 1.5T or 3T due to the shorter $T_{1}$ of fat. Moreover, the reduction in chemical shift (i.e., water‐fat off‐resonance frequency) affects spectrally selective RF pulses, which are generally used for fat suppression.

In this study, we propose a highly‐efficient free‐breathing isotropic resolution 3D whole‐heart CMRA sequence with 3× undersampled balanced steady‐state‐free‐precession (bSSFP) encoding at 0.55T. Two‐dimensional (2D) iNAVs are acquired to enable 100% respiratory scan efficiency and nonrigid motion compensated image reconstruction. Patch‐based low‐rank denoising is employed to accelerate the scan and account for the reduced SNR at 0.55T. Given the complex relationship between imaging parameters and their effects on SNR and fat suppression, sequence parameters were optimized through a combination of Bloch simulations and in vivo experiments. In vivo evaluation in healthy subjects was performed to validate the performance of the proposed approach.

## 2 METHODS

### 2.1 Acquisition sequence

**FIGURE 1:**
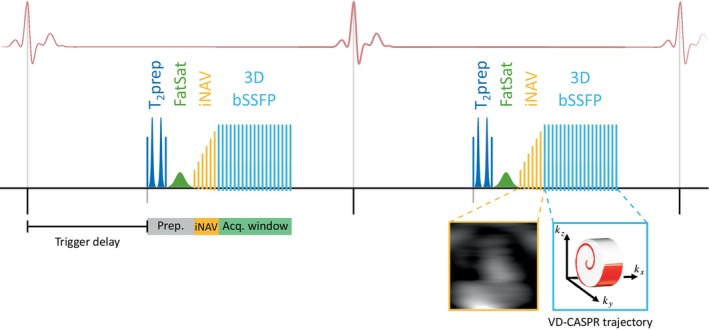
Proposed cardiac magnetic resonance angiography (CMRA) sequence at 0.55T. $T_{2}$ preparation ($T_{2}$ prep duration of 50 ms) and fat saturation (FatSat flip angle of 180°) prepulses are employed to improve visualization of the myocardium, coronary arteries, and great vessels. Two‐dimensional (2D) image navigators (iNAVs) are included to estimate 2D translation respiratory motion correction at each heartbeat, enabling beat‐to‐beat translational and bin‐to‐bin non‐rigid respiratory motion correction. Three‐dimensional (3D) whole‐heart CMRA acquisition was performed with 3× accelerated balanced steady‐state‐free‐precession (bSSFP) with a variable‐density Cartesian trajectory with spiral‐like profile order (VD‐CASPR). (A) Effect of heart rate and flip angle in tissue contrast; (B) Effect of off‐resonance and FatSat flip angle in fat signal.

The proposed non‐CE whole‐heart CMRA sequence at 0.55T (Figure 1) is based on previously proposed whole‐heart CMRA at 1.5T.^34^ It consists of an ECG‐triggered free‐breathing iNAV‐based motion‐corrected 3D bSSFP research sequence with undersampled variable density spiral‐like Cartesian (VD‐CASPR) trajectory.^35^ A 2D iNAV^25^  precedes the imaging sequence to enable respiratory binning of the 3D data, intrabin translational respiratory motion correction, and bin‐to‐bin nonrigid motion‐corrected reconstruction^26^ (further described in Section 2.3). A subject‐dependent acquisition window was positioned at mid‐diastole to minimize cardiac motion. Trigger delay and acquisition window were determined from a transversal free‐breathing cine scan performed immediately before the whole‐heart sequence. $T_{2}$ preparation ($T_{2}$prep) and fat saturation (FatSat) prepulses are applied every heartbeat to maximize contrast between blood and myocardium and to suppress epicardial fat. The $T_{2}$prep used hyperbolic‐secant adiabatic‐refocusing RF pulses, to avoid problems with fat off‐resonance at the coronaries, and the spectrally selective FatSat pulse used a Gaussian pulse shape.

Sequence parameter optimization is described in Section 2.2. Main acquisitions parameters included: field of view (FOV) = $312\times 312\times 120\;{\text{mm}}^{3}$, spatial resolution = 1.5 mm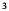, imaging flip‐angle = 110°, TE/TR = 2.65/5.3 ms, $T_{2}$ prep duration = 50 ms, FatSat flip‐angle = 180°, 3× acceleration, phase oversampling (right‐left) of 30%, slice oversampling (anterior–posterior) of 20%, coronal orientation, no saturation bands, and slab‐selective readouts, resulting in a total scan time of ≈6 min. The FOV was positioned so the slab‐selective pulses minimized inclusion of fat present in the chest wall.

### 2.2 Sequence parameter optimization

Bloch simulations were performed using KomaMRI.jl^36^ to optimize the proposed whole‐heart CMRA parameters. Two simulation experiments were performed, (1) to optimize the imaging flip angle, and (2) to optimize the FatSat flip angle. Both experiments were performed with a set $T_{2}$ prep duration of 50 ms. Each tissue was represented with 200 isochromats distributed along the z‐axis to simulate gradient spoiling effects. The isochromats for each tissue were inside a one‐dimensional voxel of size 1.5 mm. The values for $T_{1}$ and $T_{2}$ for blood, myocardial muscle, and fat at 0.55T were obtained from the work of Campbell‐Washburn, et al.^31^ Fat spins were simulated using a chemical shift of −3.4 ppm, simulating regular fat with $T_{1}=183\;\text{ms}$, and fast‐recovering fat with $T_{1}=130\;\text{ms}$. Three heartbeats were considered to approximately achieve steady‐state magnetization and the fourth heartbeat was used to measure the magnetization results. The sequence was based on Figure 1, replacing all RF pulses with hard pulses, but the FatSat pulse remained a Gaussian soft pulse.

Special care is required when simulating the FatSat pulse at 0.55T, as its duration ($T_{\text{RF}}=26.6\;\text{ms}$) is not negligible compared to the fat signal recovery at this field strength. The fat spins will experience considerable $T_{1}$ recovery during the FatSat pulse. Therefore, simplifying this prepulse to a block pulse with flip angle α would lead to incorrect conclusions.

For long RF waveforms the “flip angle” (α) does not match the tip angle experienced by the spins, as the magnetization is actively recovering during excitation. Consequently, the FatSat's flip angle is defined by:

$$\alpha =\gamma \int_{0}^{T_{\text{RF}}}B_{1}(t)\;dt,$$

where γ is the gyromagnetic ratio and $B_{1}(t)$ represents the RF pulse amplitude in the rotating frame.

For the first simulation experiment, SNR was maximized by varying the imaging flip angle between 20° and 180°. To optimize SNR independently of heart rate, multiple heart rates (ranging from 55 and 85 bpm) were simulated, and the mean and standard deviation of the obtained blood signal were calculated. Myocardial tissue was also simulated using the same procedure, and the contrast between blood and myocardial signal was computed.

For the second simulation experiment, the fat signal was minimized by varying the FatSat flip angle (between 20° and 250°) using six iNAV readouts (identified experimentally to result in good fat suppression). To be robust to $B_{0}$ inhomogeneities, multiple simulations with tissue frequency shifts (between −1 and 1 ppm, twice of what was reported by Restivo et al.^37^) were performed, and the mean and SD of the obtained fat signal were calculated.

An interactive and reproducible notebook to replicate the simulation experiments can be found in JuliaHealth.org/KomaMRI.jl/dev/tutorial‐pluto/02‐low‐field‐cmra‐optimization/.

The time between the FatSat pulse and the image acquisition needs to be minimized to prevent fat signal recovery. This can be achieved by reducing the number of iNAV readouts. Nevertheless, this could affect the steady‐state of the acquisition and the motion‐correction. To evaluate these effects, in vivo experiments were performed in one healthy volunteer varying the number of iNAV readouts (6, 10, and 14 readouts) used in the acquisition with the optimized imaging flip angle. The vessel sharpness of the proximal right coronary artery (RCA) was used in this case as a measure for image quality and fat suppression on the coronary arteries.

### 2.3 Image reconstruction and postprocessing

The images were reconstructed using a motion‐corrected iterative SENSE (itSENSE) algorithm as previously proposed at 1.5T.^26^ Briefly, this reconstruction is divided into four steps: (1) respiratory binning of 3D data, (2) beat‐to‐beat intrabin translational motion correction, (3) bin‐to‐bin nonrigid motion estimation, and (4) nonrigid motion‐corrected reconstruction. iNAVs are used to estimate 2D translational motion, enabling the assignment of 3D data to different respiratory bins. Then, data from each bin is used to reconstruct representative images per bin, which allows the estimation of nonrigid respiratory fields with respect to the end‐expiration bin. Finally, the nonrigid motion‐corrected reconstruction solves the following optimization problem:

$$\underset{x}{\min}\;\frac{1}{2}\|Ex-y{\|}_{2}^{2},$$

where x is the reconstructed image at end‐expiration, y the undersamped k‐space data after translational motion correction, and the encoding operator E is given by:

$$E=\overset{N_{\text{bins}}}{\underset{b=1}{\sum }}A_{b}FSU_{b},$$

where $A_{b}$ is the undersampling mask for bin b, F is the Fourier transform, S represents the coil sensitivities,^38^ and $U_{b}$ the nonrigid respiratory motion field for bin b. The optimization problem is solved by using a linear conjugate gradient method.^26^ After reconstruction, a locally low‐rank patch‐based (PROST) denoising^39^ is applied to further increase image quality.

Both image reconstruction and PROST denoising were performed in‐line using the Siemens ICE framework on the 0.55T MRI scanner. Image reconstruction was performed with and without PROST denoising for comparison purposes.

### 2.4 In vivo study

In vivo experiments with the proposed whole‐heart CMRA sequence were performed on 11 healthy subjects (six men and five women, age 31±4 years) on a 0.55T scanner (MAGNETOM Free.Max, Siemens Healthineers), using a six‐channel small flexible coil and nine‐channel spine coil, with an external ECG monitor (Expression MR400, Philips Healthcare). Written informed consent was obtained from all subjects prior to imaging and the study was approved by the local Institutional Review board. Acquisition parameters were the same as described in Section 2.1, with an average acquisition window of 110 ms to minimize cardiac motion.

A 3D two‐point Dixon fat reference was also acquired at end‐expiration for all volunteers for comparison purposes and to assess the quality of fat suppression with the proposed 3D whole‐heart CMRA sequence. Imaging parameters included: 3D breath‐held no‐ECG gated CAIPIRINHA,^40^ stack of stars k‐space sampling, FOV = $320\times 250\times 240\;mm^{3}$, resolution = $0.6\times 0.6\times 3\;{\text{mm}}^{3}$, imaging flip‐angle of 20°, ${\text{TE}}_{1}/{\text{TE}}_{2}/\text{TR}=3.06/6.47/10.1\;\text{ms}$, 2×2 acceleration, resulting in a total scan time of ≈21 s.

Maximum Intensity Projections (MIPs) were generated for all subjects to evaluate the ability of the 3D whole‐heart sequence to visualize the major vessels in the thorax including lung and liver vessels.

Image qualitative assessment was performed by a clinical expert (European Association of Cardiovascular Imaging level 3 accreditation, 4 years of experience). A 5‐point scoring system was used to assess all intrapericardiac structures, evaluating sharpness of vessel or cardiac wall borders (from 1 = nondiagnostic to 5 = excellent) and robustness to artifacts (from 1 = severe artifacts to 5 = minimal artifacts).

Quantitative assessment was performed by measuring the vessel sharpness of the first 4 cm of RCA using the software Soap‐Bubble,^41^ which calculates the Deriche first‐order derivative normal to the coronary segment. A vessel sharpness of 100% indicates a maximum signal intensity change at the vessel border. Average measurements were compared against previous reported studies at 1.5T.

## 3 RESULTS

### 3.1 Sequence parameter optimization

**FIGURE 2:**
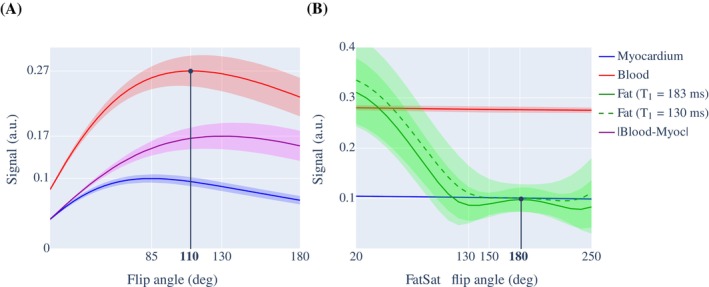
Optimization of imaging parameters for the proposed three‐dimensional whole‐heart cardiac magnetic resonance angiography at 0.55T. (A) The obtained imaging flip angle that maximizes blood signal (solid red line) was $110^{\circ }$. The SD of the measurements shows the variability with respect to heart rate. (B) The obtained FatSat flip angle that minimized the fat signal (solid green line), fast recovering fat (dashed green line), and their SD was $180^{\circ }$. The SD of the measurements shows the variability with respect to off‐resonance, to test the robustness of the pulse to variations in the $B_{0}$ field. The fat simulations used the optimized flip angle of $110^{\circ }$ produced in (A).

The simulation results for the imaging and FatSat flip angle are shown in Figure 2. The imaging flip angle that maximized blood signal (red) was found to be $110^{\circ }$. This imaging flip angle was close to the flip angle $130^{\circ }$, which maximized the blood‐myocardium contrast (purple). Sensitivity to heart rate variation is reported as the SD of the simulated signal in Figure 2A.

The FatSat flip angle that minimized signal from regular fat (solid green line) and fast‐recovering fat (dashed green line) was found to be $180^{\circ }$. Sensitivity to off‐resonance is reported as the standard deviation of the simulate fat signal in Figure 2B.

Due to RF system limitations with the RF amplifier, the maximum achievable imaging flip angle for this sequence's RF pulse was $\alpha_{\max}=80^{\circ }$. To achieve the optimal flip angle of $110^{\circ }$ (Section 3.1), the duration of the imaging RF pulses was therefore extended accordingly (from 1 to 1.4 ms, preserving the time‐bandwidth product).

**FIGURE 3:**
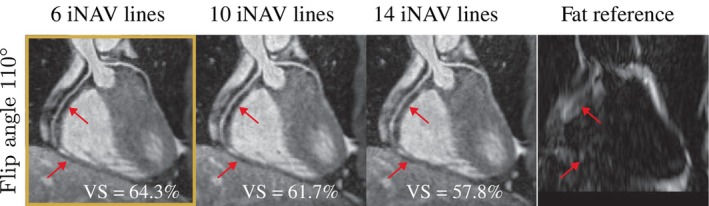
Optimization of number of Image navigator (iNAV) readouts for three‐dimensional (3D) whole‐heart cardiac magnetic resonance angiography at 0.55T. A reduction in Vessel Sharpness (VS) is observed as the number of iNAV readouts increases. This is explained due to the shorter $T_{1}$ of epicardial fat, recovering faster, and therefore decreasing myocardial and coronary vessel contrast.

The number of iNAV readouts influenced the epicardial fat suppression and subsequently the quality of the resulting CMRA images as it affects the time delay between the FatSat pulse and the center of k‐space of the imaging sequence. This is explained due to the shorter $T_{1}$ of epicardial fat, recovering faster, and therefore decreasing myocardial and coronary vessel contrast. Figure 3 shows how the fat suppression improves and the vessel sharpness increased as the time delay between the FatSat pulse and the imaging sequence is reduced, without noticeable effects in the steady‐state signal or motion correction performance. Six iNAV readouts were therefore used for the proposed whole‐heart CMRA at 0.55T, which is lower than the number of readouts used at 1.5T.

### 3.2 Image reconstruction and post‐processing

**FIGURE 4:**
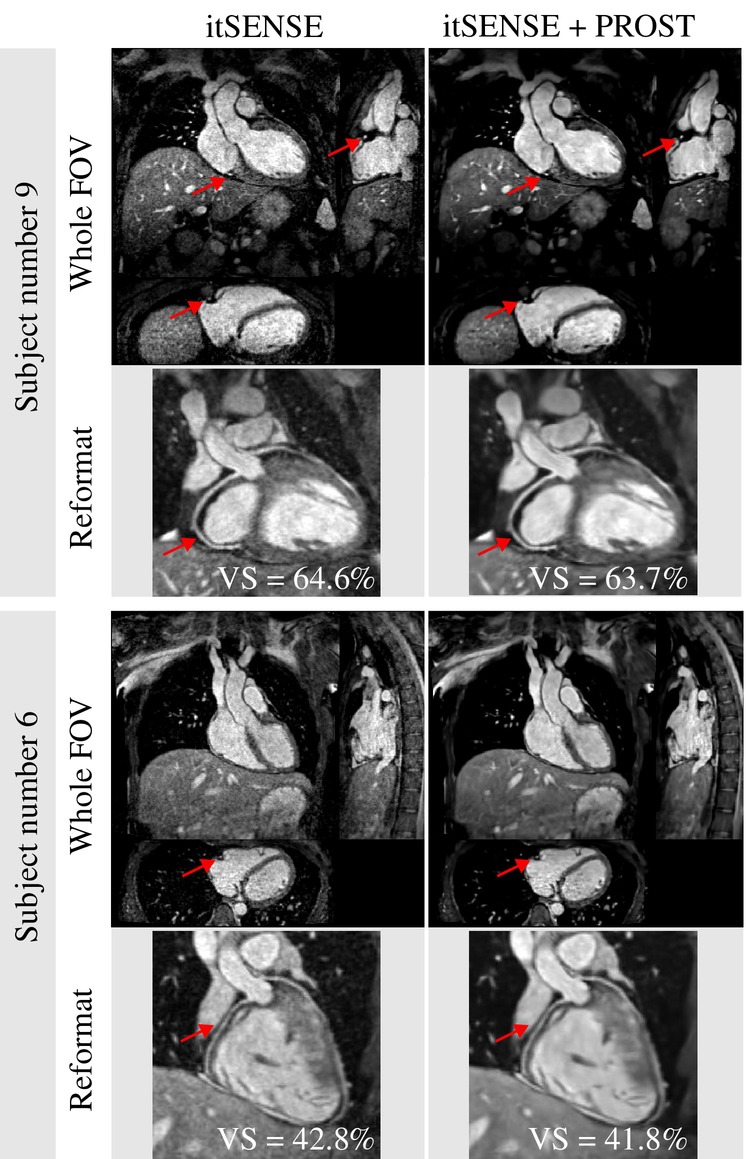
Motion‐corrected itSENSE reconstruction with (Right) and without (Left) PROST denoising. All the images are displayed in three orthogonal views, and a reformatted view. The PROST denoising improved image quality with a small detriment in vessel sharpness.

Both image reconstruction and PROST denoising were performed in‐line in the 0.55T MRI scanner. The total image reconstruction was ≈10 min (≈5 min non‐rigid motion‐corrected reconstruction and ≈5 min PROST denoising). Reconstruction for the proposed 3D whole‐heart CMRA with and without PROST denoising is shown in Figure 4. The use of PROST denoising improved visual image quality at a slight cost in vessel sharpness (itSENSE: 51.4%±10.8% vs itSENSE+PROST: 50.3%±9.8%, p‐value of 0.054).

### 3.3 In vivo study

All healthy subjects' scans were successfully completed. Acquisition time was 5.9±0.7 min. Average heart rate across all subjects was 60.3±10.8 bpm.

**FIGURE 5:**
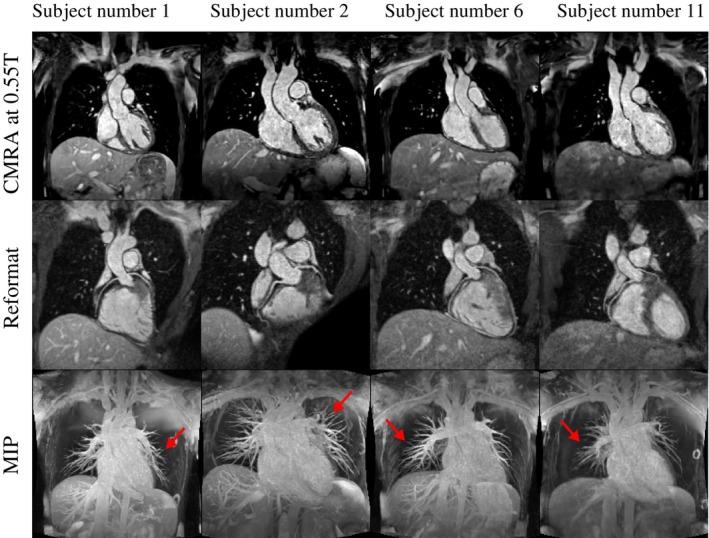
Results for the proposed whole‐heart cardiac magnetic resonance angiography (CMRA) at 0.55T for four representative healthy subjects. Coronal (top), reformatted view displaying the proximal right and left coronary arteries (middle), and Maximum Intensity Projections (MIPs) (bottom) are included. The red arrows point to the pulmonary veins, which typically exhibit banding artifacts in balanced steady‐state‐free‐precession (bSSFP) sequences at higher field strengths.

Results for four representative healthy subjects are shown in Figure 5 for coronal view, reformatted view displaying the proximal right and left coronary arteries and corresponding MIPs. Results show excellent visual image quality for all subjects. The proximal coronary arteries are well visualized in the reformatted view for all subjects. MIPs demonstrate good visualization of the heart and pulmonary veins which are often affected by off‐resonance in bSSFP sequences at higher field strengths.^42^

**FIGURE 6:**
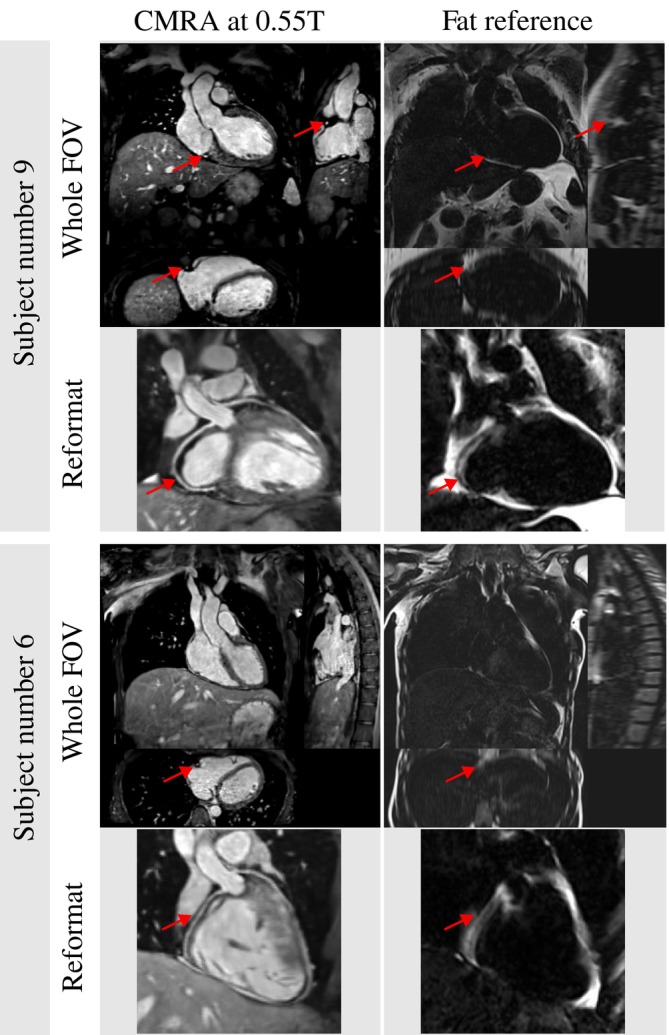
Results for the proposed whole‐heart cardiac magnetic resonance angiography (CMRA) at 0.55T for two representative healthy subjects compared to the Dixon‐fat reference. For each subject, three orthogonal views are displayed (top), and a reformatted view displaying the proximal right and left coronary arteries is also included (bottom). The red arrows point to the right coronary artery in different views, highlighting its location, which is surrounded by fat tissue.

Results for two representative healthy subjects are shown in Figure 6 in comparison to the Dixon‐fat reference. Images are shown in three orthogonal views, and a reformatted view. Results showed that fat suppression was effective with the proposed sequence, especially in the shimmed areas around the heart (see reformat), but some remaining fat signal can still be seen outside of shimmed areas close to the subclavian veins (see orthogonal views).

Image quality scores for all intrapericardiac structures are shown in Table 1 in terms of image sharpness and presence of artifacts. Excellent image sharpness (4.9±0.2 in average for all structures) and a minimal presence of artifacts (4.9±0.2 in average for all structures) was observed across all subjects.

**TABLE 1:** Average qualitative scores for all 11 healthy subjects in all intrapericardiac structures.

Structure	Sharpness	Artifacts
Left pulmonary vein	4.8	4.7
Right pulmonary vein	4.8	4.7
Left atrium	4.9	5.0
Right atrium	5.0	5.0
Left ventricle	5.0	5.0
Right ventricle	5.0	5.0
Ascending aorta	5.0	5.0
Main pulmonary artery	5.0	5.0
Superior vena cava	5.0	5.0
Inferior vena cava	5.0	5.0
Left main stem	4.9	4.8
Right coronary artery	4.9	4.8

The RCA vessel sharpness measured on the proposed whole‐heart CMRA without PROST denoising was on average 51.4%±10.8%, which is comparable to results obtained at higher field strengths (56.7%±9.6% at 1.5T without PROST).^34^ The vessel sharpness measured on the proposed whole‐heart CMRA with PROST denoising was slightly lower at 50.3%±9.8% (p‐value of 0.054).

## 4 DISCUSSION

In this study, a highly efficient free‐breathing non‐CE 3D whole‐heart MR angiography sequence was developed and evaluated at 0.55T. The proposed approach enabled 3D whole‐heart CMRA at 0.55T in a ≈6‐min scan (5.9±0.7 min), providing excellent image quality, minimal artifacts, and comparable vessel sharpness to previous studies at 1.5T.

This was achieved based on an iNAV‐based nonrigid motion corrected 3D whole‐heart CMRA framework previously proposed for 1.5T ($90^{\circ }$ imaging flip angle, $130^{\circ }$ FatSat flip angle, and 14 iNAV readouts). The framework was optimized for 0.55T by (1) increasing the bSSFP flip angle to $110^{\circ }$ in order to compensate for the loss in SNR, by (2) optimizing the FatSat parameters including increasing the FatSat flip angle to $180^{\circ }$ and shortening the time delay between the used spectrally selective fat suppression pulse and the center of k‐space of the subsequent bSSFP data acquisition, which was achieved by reducing the number of iNAV readouts, and (3) by enabling in‐line nonrigid motion‐corrected reconstruction and PROST denoising to accelerate the scan and address the reduced SNR at 0.55T.

A FatSat flip angle of $180^{\circ }$ was found to be the most effective in fat suppression. It is important to clarify that this does not mean inversion was achieved; instead, as explained in Section 2.2, the flip angle here is a representation of the $B_{1}$ amplitude. In practice, residual fat signal from the chest wall and arms (nor shimmed areas) were still observed (MIPs in Figure 5) but minimized by the use of a small anterior six‐channel coil.

Other fat saturation techniques, such as Short‐Tau Inversion Recovery and SPectral Attenuated Inversion Recovery, could enhance fat suppression across the entire FOV and will be investigated in future work. Additionally, pulse‐shape optimization, like Shinnar–Le Roux^43^ optimized pulses, might be effective in avoiding water signal suppression at high flip angles and could be investigated in the future.

The number of iNAV readouts also influenced epicardial fat suppression and thus the quality of the resulting CMRA images as it affects the time delay between the FatSat pulse and the center of k‐space of the imaging sequence. The combination of six iNAV readouts and an imaging flip angle of $110^{\circ }$ was chosen, based on simulation (Figure 2) and experimental results (Figure 3). This imaging flip angle was found to be optimal for a range of different heart rates in simulations. Similar results were observed in‐vivo in healthy subjects for heart rates in the range of 60.3±10.8 bpm. The number of iNAV readouts at 0.55T was lower than those used at 1.5T, however this did not have a visible effect in efficacy of the motion correction, with RCA sharpness measured at 0.55T being comparable to that measured at 1.5T in previous studies.

The effectiveness in fat suppression of the proposed sequence was validated by visual comparison with a Dixon fat reference, as demonstrated by the images of two representative healthy volunteers in Figure 6. Good fat suppression performance in shimmed areas around the heart is displayed by the reformatted image view, which corroborates the validity of the FatSat optimization. However, in the three orthogonal views of the entire FOV, residual fat signal outside of shimmed areas (such as in the subclavian veins) can still be observed.

A good visualization of the heart and pulmonary veins is provided by the MIPs of healthy subjects in Figure 5. At higher field strengths bSSFP sequences, like the proposed sequence, are generally affected by off‐resonance banding artifacts.^42^ These promising results corroborate the potential of low‐field MRI^31^ to image high‐susceptibility regions like the lungs and pulmonary veins using bSSFP sequences.

In this study, the PROST denoising was applied after nonrigid motion corrected reconstruction. This step improved image quality with a small detriment in vessel sharpness (VS‐RCA‐noPROST: 51.4%±10.8% vs. VS‐RCA‐PROST: 50.3%±9.8%, p‐value of 0.054). Denoising was applied here as a postprocessing step to reduce computational time, and enable in‐line implementation of both the reconstruction and denoising steps, allowing for immediate assessment of acquisition quality. A better, but more time‐consuming option, would be to include the PROST denoising as regularization step in the motion‐corrected itSENSE reconstruction, which has been shown to improve results in previous studies at 1.5T.^34^ This will however increase the reconstruction time to 15–25 min (depending on the number of ADMM iterations), as the PROST regularization needs to be performed during each ADMM iteration (3–5 times).

Different to previously proposed 3D whole‐heart CMRA at 1.5T, the proposed approach does not include saturation bands. This is due to the shorter $T_{1}$ at low‐field making saturation bands less effective and more difficult to optimize for tissues with different $T_{1}$ relaxation times. Saturation bands are commonly used clinically, and the lack of them can lead to aliasing artifacts originating from the arms. This could affect the image quality for larger patients, where the phase oversampling in the right–left direction might not be sufficient to cover the entire subject's arms. To mitigate this effect, we used a small coil with less sensitivity in the arms. Future studies should investigate more effective saturation band strategies and further optimize the motion correction framework to mitigate such artifacts.

Despite the promising results of our CMRA sequence at 0.55T, the study has some limitations. The evaluation of the sequence was performed on healthy subjects with normal heart rates (60.3±10.8 bpm), who are likely to have a smaller body habitus than patients with CVD. The absence of patient data in this study makes it impossible to compare the diagnostic value of the proposed 0.55T sequence with its 1.5T counterpart.^27^ Future work should focus on such comparative studies on healthy subjects and patients with CVD to evaluate the clinical value of the proposed CMRA sequence at 0.55T. This would provide insight into the trade‐offs and benefits of lower‐field cardiac MRI.

## 5 CONCLUSIONS

The proposed approach enables free‐breathing non‐CE 3D whole‐heart CMR angiography at 0.55T in a ≈6‐min scan, providing excellent image quality, minimal artifacts, and comparable vessel sharpness to previous studies at 1.5T. The entire reconstruction was performed in‐line in the scanner software.

Future work will include the evaluation of the proposed approach in patients with CVD and comparison against 1.5T in the same cohort of healthy subjects in terms of diagnostic value.

## CONFLICT OF INTEREST STATEMENT

K. P. Kunze is an employee of Siemens Healthcare Limited.

## Data Availability

The simulations done in this work are available as a reproducible Pluto notebook at JuliaHealth.org/KomaMRI.jl/dev/tutorial‐pluto/02‐low‐field‐cmra‐optimization/. To facilitate the reproduction of results, we also provide a Binder link, which eliminates the need of a local installation. Although it can take longer to launch and run (local: 2 min vs. Binder: 6 min). Checking the “Status/Notify when done” checkbox after clicking “Run notebook code” can be handy. Due to our institution's ethical guidelines, we cannot publish the volunteers' images. However, we are happy to share the data upon reasonable request.
